# A Pilot Study on the Urine Proteome of Cats Fed a High-Protein Complete Diet, Supplemented with or without Arginine, Ornithine or Zeolite

**DOI:** 10.3390/vetsci9120654

**Published:** 2022-11-22

**Authors:** Nadine Paßlack, Katharina Nöbauer, Karin Hummel, Ebrahim Razzazi-Fazeli, Vitaly Belik, Jürgen Zentek

**Affiliations:** 1 Institute of Animal Nutrition, Freie Universität Berlin, 14195 Berlin, Germany; 2 VetCore Facility for Research, University of Veterinary Medicine, 1210 Vienna, Austria; 3 Institute for Veterinary Epidemiology and Biostatistics, Freie Universität Berlin, 14163 Berlin, Germany

**Keywords:** urine, feline, proteome, liquid chromatography coupled to mass spectrometry, nutrition

## Abstract

**Simple Summary:**

The number and kind of proteins in the urine are of increasing scientific interest, as they might provide information on the health status of an organism. Besides diseases, however, further factors could influence the so-called “urine proteome”, such as dietary intake. In the present investigation, the urine of four healthy adult cats was analyzed. Each cat received a high-protein diet without or with supplements that could affect the protein metabolism: arginine, ornithine or zeolite. At the end of the feeding periods, the urine of the animals was collected. We found a huge number of proteins in the urine of the cats (means between 399 and 516 urinary proteins). According to an on-line database search, these proteins are associated with several biological processes. When the feeding groups were compared, a higher or lower abundance of some urinary proteins could be observed in the cats receiving the dietary supplements than the control treatment. Overall, our study provides basic data on the urine proteome of healthy adult cats. The huge number of urinary proteins implies the potential to identify biomarkers for health and disease, as well as for metabolic processes, which should be further investigated in future studies.

**Abstract:**

Proteome analyses can be used to detect biomarkers for the healthy and diseased organism. However, data in cats are scarce, and no information is available on the potential impact of nutritional interventions on the feline urine proteome. In the present study, a label-free shotgun proteomics approach was performed to investigate the urinary proteins of four healthy adult cats. Each animal received a high-protein complete diet without (w/o) or with supplements that could affect the protein metabolism: arginine (+100% compared to the arginine concentration in the w/o diet), ornithine (+200% compared to the arginine concentration in the w/o diet) or zeolite (0.375 g/kg body weight/day). Our results demonstrate a huge number of proteins in the urine of cats (516 ± 49, 512 ± 39, 399 ± 149 and 455 ± 134 in the w/o, arginine, ornithine and zeolite group, respectively), which are associated with several biological processes. In addition, up- and downregulated urinary proteins could be detected in the dietary supplementation periods. Overall, the present pilot study provides basic data on the urine proteome of healthy adult cats. With increasing information, the numerousness of urinary proteins implies the potential to identify biomarkers and metabolic pathways in the feline organism.

## 1. Introduction

Urine is an important biological test material for nutritional research as well as medical diagnostics. It is advantageous due to its non-invasive, painless collection, and contains substances that provide information on the health status of the organism [1]. In this context, urinary proteome analyses are of increasing interest, as specific proteins have been demonstrated to be associated with several diseases [1]. These studies, however, have predominantly been performed in human medicine research, while investigations related to veterinary medicine are relatively scarce. To our best knowledge, only three studies have been published on the urine proteome of cats so far, with their focus especially on animals with chronic kidney disease [2,3] and urinary tract diseases [4]. Jepson et al. [2] found differences in the urine proteome clusters of cats with a chronic kidney disease that developed either azotemia within one year after sample collection or remained nonazotemic. In this study, surface-enhanced laser desorption–ionization time-of-flight mass spectrometry (SELDI-TOF-MS) was used for the proteome analyses [2]. Ferlizza et al. [3] compared the urine proteome of healthy cats and cats with a chronic kidney disease (IRIS stage 2–4). They observed 13 over- or underrepresented proteins in the urine of the kidney patients compared to the control animals, making them potential candidates as biomarkers of renal function [3]. The methods used for this investigation included one-dimensional sodium-dodecyl-sulfate polyacrylamide gel electrophoresis and two-dimensional electrophoresis (2DE), followed by mass spectrometry [3]. Finally, Lemberger et al. [4] evaluated the urine proteome of healthy control cats and cats with idiopathic cystitis, bacterial urinary tract infection and urolithiasis, using 1-dimensional gel electrophoresis. The urinary fibronectin concentrations were further evaluated by Western blot and immunohistochemical measurements [4]. The authors found that the urinary fibronectin concentrations were higher in the cats with idiopathic cystitis when compared to the control cats, and assumed that fibronectin could, therefore, act as a potential biomarker for this disease [4]. In addition, the protein concentrations in the urine of the cats with urinary tract diseases were higher than in the urine of the control animals, and patients with urolithiasis had higher urinary protein concentrations compared to cats with idiopathic cystitis [4].

Although the available studies have focused on the effects of diseases on the urine proteome of cats, dietetic interventions might also be an influencing factor, as nutrition substantially modulates metabolic pathways [5]. Several nutritional factors might be of interest for proteomic research, among others the protein or amino acid supply. For the present investigation, we used urine samples of a study evaluating the impact of a high-protein diet supplemented without or with arginine, ornithine and zeolite in healthy adult cats [6]. As the main findings of this study, the postprandial blood urea increase in the animals was enhanced by the dietary arginine and ornithine supplementation, but the renal urea excretion did not increase simultaneously. Thus, it can be speculated that the supplements promoted the hepatic ammonia detoxification in felines, but that the renal excretory mechanisms might have been impaired by the dietetic interventions. In addition, the dietary zeolite supplementation seemed to affect the nitrogen metabolism as well, as demonstrated by decreased concentrations of biogenic amines in the feces of the cats and a reduced renal ammonia excretion [6]. In order to evaluate the impact of the dietary supplements in the feline organism in more detail, we aimed to perform a proteomics approach, using the urine of the animals. We hypothesized that the analyses would reveal a dietary modulation of the cats’ protein metabolism or kidney function. The objectives of the present study were, therefore, two-fold: (I) to generate basic data on the urine proteome of healthy adult cats, and (II) to assess the effects of a dietary arginine, ornithine and zeolite supplementation on the feline urine proteome.

## 2. Materials and Methods

The study was approved by the relevant authority in Berlin, Germany (Landesamt für Gesundheit und Soziales; approval number G 0120/15).

The design of the feeding study has been previously published in detail [6]. In the present investigation, urine samples of a part of this study were used for the proteome analyses.

### 2.1. Feeding Study

Four healthy adult cats (2 male neutered, 2 female intact, aged 80.3 ± 20.8 months at the beginning of this study) received a high-protein basic diet (60.3% crude protein in dry matter) supplemented without (w/o) or with arginine (+100% compared to the arginine concentration in the basic diet), ornithine (+200% compared to the arginine concentration in the basic diet) or zeolite (0.375 g/kg body weight/day) for 11 days each. On the last four days of the feeding periods, the cats were housed in metabolic cages to collect urine.

### 2.2. Proteome Analyses

For the label-free quantitative shotgun proteomic approach, the concentration of total protein in the urine samples was determined using the Pierce™ 660 nm Protein Assay Reagent (Thermo Fisher Scientific, Waltham, MA, USA), following the manufacturer’s protocol. Different volumes of urine, corresponding to 30 µg protein, were filled up to 500 µL with 8 M urea in 50 mM TRIS (both Roth, Karlsruhe, Germany). These samples were loaded onto a Pall 10 kDa filter (Pall Corporation, Port Washington, NY, USA) for a FASP digest and subsequent liquid chromatography coupled to mass spectrometry (LC-MS) analysis, as described by Soler et al. [7]. In brief, the digested protein extracts were desalted using C18 spin columns (Pierce, Thermo Fisher Scientific), dried in an Eppendorf concentrator plus (Eppendorf, Hamburg, Germany) and solved in 0.1% TFA (Thermo Fisher Scientific) to a peptide concentration of 0.1 µg/µL. The separation of 300 ng of the peptides was performed on a 25 cm Acclaim PepMap C18 column (75 µm inner diameter, 2 µm particle size and 100 Å pore size) with a flow rate of 300 nL/min on a Thermo nano-HPLC system, coupled to a Q Exactive HF Orbitrap mass spectrometer (both Thermo Fisher Scientific) using a 60 min gradient [7]. The LC-MS analyses were performed in triple determination per sample.

### 2.3. Database Searches

For a first evaluation of the proteome data, the Proteome Discoverer Software 2.4.0.305 (Thermo Fisher Scientific) was used with the following settings: Protein database: NCBI_cat_tx9685_190612.fasta; accessed on 12 June 2019; crap.fasta (https://www.thegpm.org/crap/; accessed on 20 November 2016); enzyme name: trypsin (full); max. missed cleavage sites: 2; precursor mass tolerance: 10 ppm; fragment mass tolerance: 0.02 Da; dynamic modification: oxidation/+15.995 Da (M); N-terminal modification: Acetyl/+42.011 Da (N-terminus); static modification: carbamidomethyl/+57.021 Da (C); decoy database search: target FDR (strict): 0.01; target FDR (relaxed): 0.05; validation based on: q-value.

After bioinformatical analysis, the number of identified proteins in the urine samples could be determined for the three LC-MS injections. The accessions of the identified proteins were then submitted to the STRING database (https://string-db.org; accessed from 17 March 2022 to 11 September 2022), which was performed separately for each cat and each feeding group. The settings for the database search were: multiple proteins; list of names: protein accession; organisms: *Felis catus*.

As the identified proteins of the three injections were submitted together to the STRING database, doublings were removed by the system, resulting in the output of all urinary proteins found for one cat per feeding group. The urinary proteins of the cats of the same feeding group were again submitted to the STRING database to receive all urinary proteins found for each feeding group.

### 2.4. Biological Processes

The urinary proteins are associated with several biological processes, as specified by the STRING database. In a first step, the biological processes were summarized to the following groups: immune/stress response; cell communication/activity; nutrient metabolism and catabolic processes; tissue/organ development, cell structure and wound healing; blood coagulation, fibrinolysis and hemostasis; others. This analysis was a qualitative evaluation, i.e., if a biological process was found for different cats of the same feeding group, it was counted as one. In a second step, the number of proteins associated with a biological process was considered to compare the feeding groups.

### 2.5. KEGG (Kyoto Encyclopedia of Genes and Genomes) Pathways

The up- and downregulated urinary proteins between the arginine, ornithine or zeolite group and the w/o group (see statistical data analyses) were submitted to the STRING database (search for multiple proteins, list of names: protein accession, organism: *Felis catus*) to detect the associated KEGG pathways.

### 2.6. Statistical Data Analyses

The full proteome data set is provided as a Supplementary Material.

A first statistical data analysis was carried out by the *t*-test (Proteome Discoverer Software 2.4.0.305, Thermo Fisher Scientific) to detect up- and downregulated proteins between the supplemented groups versus the w/o group (fold change ± 2). An FDR adjusted *p*-value < 0.05 was considered to be statistically significant. In addition, a principal component analysis was carried out in the course of the protein quantification (Proteome Discoverer Software 2.4.0.305, Thermo Fisher Scientific; formatting with Microsoft PowerPoint 2016).

The distribution of the biological processes associated with the proteins found in the urine of the dietary treatment groups was qualitatively compared by Microsoft Excel (2016). Additionally, the number of proteins associated with a single biological process was evaluated. For this, the feeding groups were again compared by the *t*-test (SPSS 28, IBM Corp., Armonk, NY, USA), with a *p*-value < 0.05 being statistically significant.

## 3. Results

### 3.1. Urinary Protein Concentration and Number of Identified Proteins

The analyzed protein concentration in the urine samples of the cats was 64.7 ± 26.2, 80.8 ± 39.4, 49.6 ± 33.0 and 53.6 ± 41.7 µg/mL for the w/o, arginine, ornithine and zeolite treatment, respectively.

The LC-MS/MS method identified 516 ± 45 (w/o group), 512 ± 18 (arginine group), 399 ± 162 (ornithine group) and 455 ± 146 (zeolite group) urinary proteins. However, as demonstrated by the high standard deviations, the numbers markedly differed among the cats within each group. When the feeding groups were compared, no differences in total protein numbers could be detected between the supplemented groups versus the w/o group.

### 3.2. Biological Processes

When the identified proteins were submitted to the STRING database, 365 proteins of the w/o group could be found for *Felis catus*, which are associated with 115 biological processes. For the arginine group, 286 proteins were found and are involved in 88 biological processes. Comparable values were observed for the ornithine group, with 260 identified proteins in 84 biological processes. In the zeolite group, 342 proteins were found in the STRING database, which are associated with 115 biological processes.

**Figure 1:**
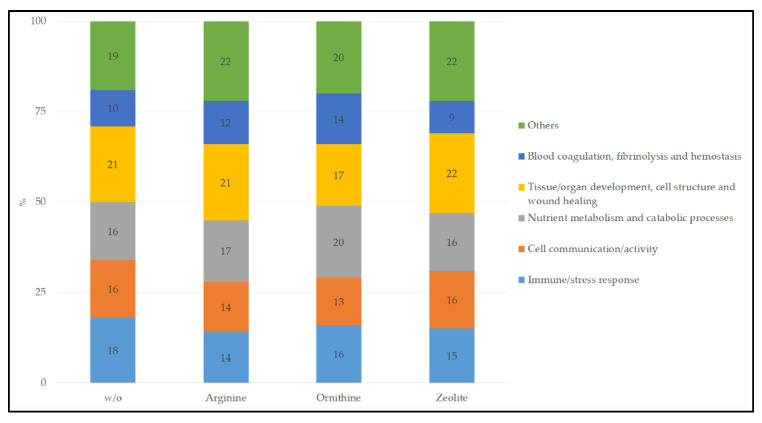
Summary of the biological processes associated with urinary proteins of cats (*n* = 4) fed a high-protein diet without (w/o) or with arginine, ornithine or zeolite. More details on the single biological processes can be found in Table 1, Table 2, Table 3, Table 4, Table 5 and Table 6.

When the biological processes were summarized to groups, a comparable distribution could be observed between the four feeding groups (Figure 1).

The mean number of urinary proteins associated with the detected biological processes is presented in Table 1, Table 2, Table 3, Table 4, Table 5 and Table 6. The comparison between the supplemented groups versus the w/o treatment revealed only marginal differences, with a lower number of urinary proteins related to a “regulation of protein metabolic process” in the ornithine group, and a higher number of urinary proteins associated with a “regulation of body fluid levels” in the arginine group.

### 3.3. Up- and Downregulated Urinary Proteins and Associated KEGG Pathways

**Figure 2:**
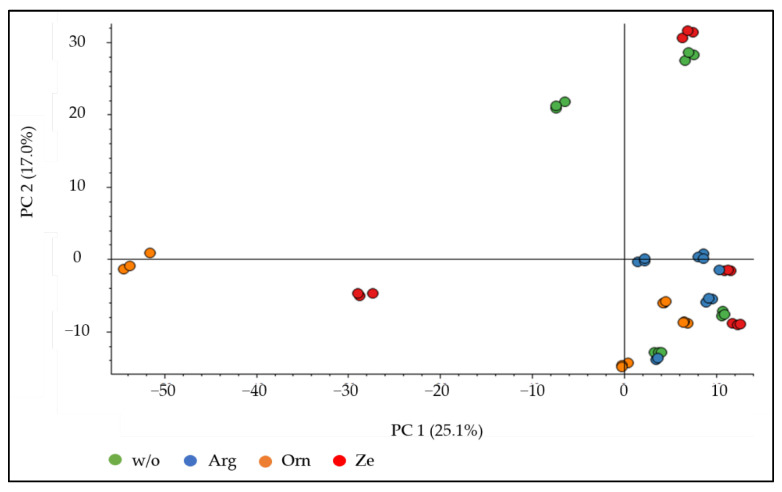
Principal component analysis plot of the urine proteome of cats (*n* = 4) fed a high-protein diet without (w/o) or with the supplementation of arginine (Arg), ornithine (Orn) or zeolite (Ze). The plot depicts the triplicate determination for each cat and feeding group.

The principal component analysis plot demonstrated a clustering of the technical replicates, but not for a specific dietary treatment (Figure 2). However, when the urine proteome of the arginine group was statistically compared with the w/o group, 80 downregulated and 27 upregulated proteins were detected. Out of the 80 downregulated proteins, 35 were found in the STRING database, where 4 proteins are associated with the KEGG pathway “glycolysis/gluconeogenesis” and 5 proteins with “*Salmonella* infection” (Table 7). Only 8 out of the 27 upregulated urinary proteins were found in the STRING database, and no associated KEGG pathways could be identified.

In the ornithine group, 125 urinary proteins were downregulated and 30 proteins were upregulated compared to the w/o group. Fifty-three of the downregulated proteins were identified in the STRING database, with 5 being associated with the KEGG pathway “Ribosome” and 5 with “*Salmonella* infection” (Table 8). Fourteen upregulated proteins were found in the STRING database, where 3 of them are related to the “Estrogen signaling pathway” (Table 8).

Eighty-five urinary proteins were downregulated and 21 urinary proteins were upregulated in the cats receiving zeolite to their diet compared to the w/o treatment. Thirty-eight of the downregulated proteins and 9 of the upregulated proteins were identified in the STRING database, but no associated KEGG pathways could be detected.

## 4. Discussion

In the present study, urine samples of cats were analyzed by a label-free quantitative approach to evaluate the feline urine proteome. While proteome analyses are especially used in human medicine to identify potential biomarkers for health and disease [1], investigations in cats remain scarce. To our knowledge, only three studies have been conducted in this context so far, focusing on cats with kidney and urinary tract diseases [2,3,4]. However, different methods were applied for the proteome analyses, some resulting in a detection of only 5 proteins [4], 17–23 protein clusters that were common to all array spectra [2], and 13–14 proteins [3] in the urine samples investigated. In the present study, the proteome approach by high resolution LC-MS/MS revealed a high number of 399–516 proteins in the urine samples of the four feeding groups, making this analytical method advantageous for an untargeted urine proteome evaluation in cats.

Nevertheless, when compared to data from human medicine, the number of detected proteins in the feline urine samples was markedly lower. Zhao et al. [8] summarized six large-scale studies on the proteome of healthy human subjects, which identified between 1310–6085 urinary proteins. The number of proteins in urine of healthy dogs ranged between 176 [9], 182 [10] and 659 [11], and is, therefore, more comparable to the data obtained for the cats of the present study. At this point, it can only be speculated why human subjects and those carnivorous species differ in their urine proteome. Besides different analytical methods used in the related studies, species-dependent differences might be relevant. In addition, individual factors, such as age, sex or dietary intake, might influence the kind and number of proteins in the urine. Research in humans is already evaluating the individual variation of the urine proteome [12] and should be explored for animal-derived samples as well, since this might facilitate data interpretation and comparison. Given the small sample size of the present pilot study, however, this aspect cannot be determined for the feline urine proteome at this stage. Nevertheless, with increasing data deriving from larger scale investigations, it can be expected that potential interfering factors on the urine proteome of cats will be identified or disproved. The present data should, therefore, be considered as a first step towards a broader knowledge of the feline urine proteome, and as a starting point for future investigations.

The urine proteome of the four feeding groups did not markedly differ. Although a notable number of down- and upregulated proteins was detected in the urine of the cats receiving the dietary supplements compared to the control treatment, the physiological relevance of this finding, as evaluated by the associated biological processes and KEGG pathways, seemed to be low. However, one interesting result was the downregulation of four proteins related to the KEGG pathway “glycolysis/gluconeogenesis” in the arginine group. It might be speculated if mechanisms related to nitric oxide were responsible for this downregulation, as nitric oxide is synthesized from arginine and can affect the glucose metabolism [13]. However, the pilot study character of the present investigation requires a careful data interpretation and follow-up studies to prove this hypothesis.

We used urine samples of a study that gained comprehensive data on the dietary effects of arginine, ornithine and zeolite on the formation and excretion of uremic toxins in cats [6]. The results indicated an impact of arginine and ornithine on the hepatic ammonia detoxification, as these supplements enhanced the postprandial blood urea increase, but also potentially detrimental effects on the renal function, since the urea excretion by the kidneys was not enhanced in parallel. In addition, effects of the dietary zeolite supplementation on the intestinal nitrogen metabolism of the cats were observed [6]. The proteome analyses of the urine samples aimed to provide more insights into the impact of the three supplements on the protein metabolism of the cats, and to identify potential indications for an impaired kidney function. However, as the proteome data were comparable among the feeding groups, including the control treatment, the results cannot support these objectives.

It should, however, be noted that the proteomics approach of the present study only allowed for a relative quantification of the urinary proteins (ratio of the identified proteins related to a given protein concentration in the samples [14]). Thus, the individual differences in the urinary protein concentrations of the cats could not be considered for data evaluation. In addition, a certain number of the identified proteins has not been found in the STRING database. This implies missing information on the detected urinary proteins, and possibly also unrecognized differences between the feeding groups. Thus, there seems to be a potential for improvements in the data sets related to the feline urine proteome. This also includes information on the associated biological processes or KEGG pathways, which were partly hard to interpret in the present investigation. For instance, some downregulated urinary proteins of the arginine and ornithine group were related to the KEGG pathway “*Salmonella* infection”, although no infection occurred in the cats enrolled in this study. It is more likely that the detected downregulated proteins are involved in more general defense mechanisms, which can, among others, be also relevant in the course of a *Salmonella* infection. Future research should, therefore, also focus on an extension of the knowledge base and analytical tools to evaluate feline urine proteome data.

In the present study, voided urine samples were used. Although it has been discussed that voided urine could be contaminated by proteins from the genitourinary tract [2], other collection methods also imply limitations. For instance, blood contamination of the urine due to cystocentesis is possible [3], which might also apply to urine collection by catheterization. In addition, both cystocentesis and catheterization are invasive and mildly stressful, making urine collection for several days to receive pooled samples difficult. These, however, can be easily obtained by voided urine samples, and might, therefore, be potentially advantageous to provide data on the average urine proteome of an animal.

## 5. Conclusions

The differences in the urine proteome of cats fed a high-protein diet with or without an arginine, ornithine or zeolite supplementation were only small, and could not yield information on a potential impact of the supplements on the feline protein metabolism or kidney function, as hypothesized based on our previous data [6]. Notwithstanding, as the proteomics approach revealed a huge number of proteins in the urine of the cats, it might be a promising tool to detect biomarkers for the healthy and diseased organism in the future. For its practical implication, however, further studies are needed to increase the knowledge on the feline urine proteome.

## Figures and Tables

**Table 1:** Urinary proteins associated with biological processes ^1^ related to the immune/stress response, detected in the urine of cats (*n* = 4) fed a high-protein diet without (w/o) or with the supplementation of arginine (Arg), ornithine (Orn) or zeolite (Ze). Mean number of proteins ± standard deviation.

Biological Process	w/o				Arg				Orn				Ze				*p*-value: w/o versus		
																	Arg	Orn	Ze
Negative regulation of humoral immune response	3			(1) ^2^				(-)	3			(1)	3			(1)	-	-	-
Acute-phase response	5			(2)	5	±	1	(4)	5	±	1	(3)	5	±	1	(3)	0.182	0.495	0.495
Acute inflammatory response	6	±	1	(4)	6	±	1	(4)	6	±	1	(3)	6			(2)	1.000	0.721	0.182
Negative regulation of response to external stimulus	12			(1)	11			(1)	11			(1)	12			(2)	-	-	0.667
Inflammatory response	15	±	1	(3)	15	±	1	(4)	13	±	1	(3)	15	±	3	(3)	0.604	0.132	0.872
Defense response	26	±	2	(3)	25	±	2	(3)	23			(2)	25	±	6	(3)	0.539	0.061	0.744
Regulation of immune system process	28	±	2	(4)	26	±	1	(4)	25	±	1	(3)	31			(2)	0.287	0.122	0.111
Response to stress	51	±	5	(3)	49	±	2	(3)	36	±	14	(3)	47	±	10	(3)	0.414	0.132	0.506
Response to stimulus	98			(1)	104			(2)	98			(2)	112			(2)	0.531	1.000	0.198
Response to other organism	26			(1)				(-)				(-)	20			(2)	-	-	0.478
Immune system process	35			(1)				(-)				(-)	36			(1)	-	-	-
Negative regulation of immune effector process				(-)				(-)	6			(1)				(-)	-	-	-
Negative regulation of immune response				(-)				(-)				(-)	7			(1)	-	-	-
Negative regulation of immune system process				(-)				(-)				(-)	13			(1)	-	-	-
Regulation of response to external stimulus				(-)				(-)				(-)	20			(2)	-	-	-
Respiratory burst				(-)				(-)				(-)	3			(1)	-	-	-
Myeloid leukocyte activation				(-)				(-)				(-)	7			(1)	-	-	-
Response to external stimulus				(-)				(-)				(-)	36			(1)	-	-	-

^1^ Gene ontology (analyzed by the STRING database); ^2^ number of positive samples.

**Table 2:** Urinary proteins associated with biological processes ^1^ related to the cell communication/activity, detected in the urine of cats (*n* = 4) fed a high-protein diet without (w/o) or with the supplementation of arginine (Arg), ornithine (Orn) or zeolite (Ze). Mean number of proteins ± standard deviation.

Biological Process	w/o				Arg				Orn				Ze				*p*-value: w/o versus		
																	Arg	Orn	Ze
Cellular oxidant detoxification	7	±	1	(3) ^2^	7	±	1	(4)	6	±	1	(3)	6	±	1	(3)	0.900	0.519	0.678
Cell-substrate adhesion	9			(2)	8	±	1	(3)	8			(2)	10			(2)	0.789	0.423	0.293
Negative regulation of cell adhesion	10			(1)	11			(1)	9			(1)	11			(2)	-	-	-
Cell adhesion	33	±	6	(4)	34	±	2	(4)	33	±	2	(3)	30	±	11	(4)	0.600	0.967	0.643
Cell-cell adhesion	17			(2)	16	±	1	(4)	15	±	2	(3)	17			(2)	0.261	0.308	0.808
Regulation of cell adhesion	19	±	1	(4)	18	±	2	(3)	19	±	1	(3)	24			(2)	0.537	0.721	0.065
Regulation of cell migration	24	±	4	(4)	24	±	2	(4)	23	±	2	(3)	28			(2)	0.904	0.664	0.190
Negative regulation of cellular process	73			(2)	69	±	4	(4)	66	±	3	(3)	76			(2)	0.512	0.258	0.590
Regulation of cell adhesion mediated by integrin	5			(1)				(-)				(-)	5			(2)	-	-	-
Cellular detoxification	8			(1)				(-)	7			(1)	8			(1)	-	-	-
Positive regulation of cell motility	18			(1)				(-)				(-)	18			(1)	-	-	-
Positive regulation of cell migration	17			(1)				(-)				(-)	17			(2)	-	-	-
Regulation of cell motility	30			(1)	22			(1)	22			(1)	29			(1)	-	-	-
Regulation of cellular component movement	27			(2)	25			(1)	27			(1)	30			(1)	0.821	1.000	0.740
Cell migration	22			(1)				(-)				(-)	20			(1)	-	-	-
Regulation of cellular component size	14			(1)				(-)				(-)	17			(1)	-	-	-
Regulation of cellular component biogenesis	24			(1)				(-)				(-)				(-)	-	-	-
Regulation of leukocyte cell-cell adhesion	10			(1)				(-)				(-)				(-)	-	-	-
Cell activation	15			(1)				(-)				(-)				(-)	-	-	-
Negative regulation of cellular metabolic process				(-)				(-)	37			(1)				(-)	-	-	-
Regulation of cell-cell adhesion				(-)				(-)				(-)	12			(1)	-	-	-
Regulation of cell activation				(-)				(-)				(-)	15			(1)	-	-	-
Regulation of cell population proliferation				(-)				(-)				(-)	28			(1)	-	-	-

^1^ Gene ontology (analyzed by the STRING database); ^2^ number of positive samples.

**Table 3:** Urinary proteins associated with biological processes ^1^ related to the nutrient metabolism and catabolic processes, detected in the urine of cats (*n* = 4) fed a high-protein diet without (w/o) or with the supplementation of arginine (Arg), ornithine (Orn) or zeolite (Ze). Mean number of proteins ± standard deviation.

Biological Process	w/o				Arg				Orn				Ze				*p*-value: w/o versus		
																	Arg	Orn	Ze
Digestion	8	±	2	(4) ^2^	7	±	1	(4)	6	±	1	(4)	7	±	1	(4)	0.555	0.146	0.537
Intestinal absorption	4			(1)				(-)				(-)				(-)	-	-	-
Digestive system process	6			(1)				(-)				(-)	4			(1)	-	-	-
Negative regulation of peptidase activity	22	±	1	(3)	24	±	2	(4)	23	±	1	(3)	21	±	4	(4)	0.229	0.492	0.693
Negative regulation of endopeptidase activity	22	±	2	(4)	23	±	2	(4)	22	±	1	(3)	20	±	4	(4)	0.445	1.000	0.473
Negative regulation of proteolysis	25	±	2	(4)	27	±	2	(4)	26	±	2	(3)	23	±	5	(4)	0.368	0.733	0.472
Regulation of peptidase activity	29	±	2	(4)	29	±	2	(4)	23	±	10	(4)	26	±	5	(4)	0.848	0.299	0.375
Regulation of endopeptidase activity	26	±	2	(4)	26	±	2	(4)	21	±	9	(4)	23	±	5	(4)	0.868	0.300	0.388
Regulation of proteolysis	32	±	2	(4)	32	±	2	(4)	26	±	11	(4)	29	±	6	(4)	0.862	0.312	0.440
Negative regulation of cellular protein metabolic process	30	±	2	(4)	31	±	2	(4)	30	±	2	(3)	26	±	7	(4)	0.533	0.739	0.347
Negative regulation of protein metabolic process	31	±	2	(4)	32	±	2	(4)	32	±	2	(3)	28	±	7	(4)	0.533	0.569	0.390
Proteolysis	29	±	2	(3)	27	±	2	(3)	26			(2)	28			(2)	0.279	0.077	0.402
Regulation of cellular protein metabolic process	49			(2)	48	±	1	(3)	44	±	2	(3)	48			(2)	0.904	0.539	0.937
Regulation of protein metabolic process	55			(1)	49			(2)	45	±	2	(3)	49			(2)	0.179	**0.028**	0.179
Negative regulation of nitrogen compound metabolic process				(-)				(-)	35			(1)				(-)	-	-	-
High-density lipoprotein particle remodeling				(-)				(-)				(-)	3			(1)	-	-	-
Glycolytic process	6			(1)				(-)				(-)	6			(1)	-	-	-
Carbohydrate metabolic process	17	±	2	(3)				(-)	14			(1)	16			(2)	-	0.270	0.599
Aminoglycan metabolic process				(-)				(-)	6			(1)				(-)	-	-	-
Monosaccharide biosynthetic process				(-)				(-)				(-)	4			(1)	-	-	-
Lipid metabolic process				(-)				(-)				(-)	15			(1)	-	-	-
Catabolic process	38			(1)				(-)	34			(1)	37			(2)	-	-	0.667
Hydrogen peroxide catabolic process	4			(1)	4			(2)	4			(1)	4			(1)	-	-	-
Ceramide catabolic process				(-)	4			(2)	5			(1)	4			(1)	-	-	-
Glycosphingolipid catabolic process				(-)				(-)	4			(1)				(-)	-	-	-
Lipid catabolic process				(-)				(-)	11			(1)				(-)	-	-	-

^1^ Gene ontology (analyzed by the STRING database); ^2^ number of positive samples.

**Table 4:** Urinary proteins associated with biological processes ^1^ related to tissue/organ development, cell structure and wound healing, detected in the urine of cats (*n* = 4) fed a high-protein diet without (w/o) or with the supplementation of arginine (Arg), ornithine (Orn) or zeolite (Ze). Mean number of proteins ± standard deviation.

Biological Process	w/o				Arg				Orn				Ze				*p*-value: w/o versus		
																	Arg	Orn	Ze
Positive regulation of response to wounding	7	±	1	(4) ^2^	7	±	1	(4)	6	±	2	(4)	7	±	2	(4)	1.000	0.595	0.811
Positive regulation of wound healing	6	±	1	(4)	6	±	1	(4)	6	±	0	(3)	6	±	2	(3)	1.000	0.638	0.814
Regulation of wound healing	11	±	1	(4)	10	±	1	(4)	10	±	2	(4)	10	±	2	(4)	0.730	0.488	0.560
Regulation of response to wounding	12	±	1	(4)	11	±	1	(4)	11	±	2	(4)	11	±	2	(4)	0.730	0.488	0.560
Wound healing	17	±	2	(4)	18	±	1	(4)	16	±	4	(4)	17	±	3	(4)	0.468	0.524	0.809
Growth plate cartilage chondrocyte differentiation	4			(1)				(-)				(-)				(-)	-	-	-
Growth plate cartilage development	5			(1)	4			(2)				(-)				(-)	-	-	-
Cornification	5			(2)	5			(2)	5			(2)	4			(1)	-	0.423	-
Skin development	12			(2)	11	±	0	(3)	10	±	3	(3)	10			(2)	-	0.302	0.126
Tissue development	37			(2)	33	±	1	(3)	31			(2)	35			(1)	0.578	0.380	0.879
Keratinization	6			(1)				(-)				(-)				(-)	-	-	-
Keratinocyte differentiation	8			(1)	7			(2)	7			(1)	7			(1)	-	-	-
Response to woundig	18			(1)				(-)				(-)				(-)	-	-	-
Regulation of actin filament polymerization	10			(1)				(-)				(-)	13			(1)	-	-	-
Epidermal cell differentiation	9			(1)				(-)	8			(1)				(-)	-	-	-
Epidermis development	11			(1)	10			(1)	10			(1)				(-)	-	-	-
Supramolecular fiber organization	17			(1)				(-)				(-)	17			(1)	-	-	-
Regulation of anatomical structure size	15			(1)				(-)				(-)	18			(1)	-	-	-
Actin filament-based process	19			(1)				(-)				(-)	20			(1)	-	-	-
Actin cytoskeleton organization	17			(1)				(-)				(-)	18			(1)	-	-	-
Response to wounding	15			(1)				(-)				(-)	16			(1)	-	-	-
Tube development				(-)	19			(1)				(-)	20			(1)	-	-	-
Collagen fibril organization				(-)	5			(1)				(-)				(-)	-	-	-
System development				(-)				(-)	61			(1)				(-)	-	-	-
Arp2/3 complex-mediated actin nucleation				(-)				(-)				(-)	5			(1)	-	-	-
Barbed-end actin filament capping				(-)				(-)				(-)	4			(1)	-	-	-
Actin nucleation				(-)				(-)				(-)	6			(1)	-	-	-
Positive regulation of actin filament polymerization				(-)				(-)				(-)	7			(1)	-	-	-
Regulation of actin cytoskeleton organization				(-)				(-)				(-)	14			(1)	-	-	-
Regulation of supramolecular fiber organization				(-)				(-)				(-)	14			(1)	-	-	-
Tube morphogenesis				(-)				(-)				(-)	17			(1)	-	-	-
Epithelium development				(-)				(-)				(-)	22			(1)	-	-	-
Anatomical structure morphogenesis				(-)				(-)				(-)	40			(1)	-	-	-

^1^ Gene ontology (analyzed by the STRING database); ^2^ number of positive samples.

**Table 5:** Urinary proteins associated with biological processes ^1^ related to blood coagulation, fibrinolysis and hemostasis, detected in the urine of cats (*n* = 4) fed a high-protein diet without (w/o) or with the supplementation of arginine (Arg), ornithine (Orn) or zeolite (Ze). Mean number of proteins ± standard deviation.

Biological Process	w/o				Arg				Orn				Ze				*p*-value: w/o versus		
																	Arg	Orn	Ze
Negative regulation of fibrinolysis	3			(2) ^2^	3			(2)	3			(2)	3	±		(1)	-	-	-
Fibrinolysis	4	±	1	(4)	4	±	1	(4)	4	±	1	(3)	3	±	1	(3)	1.000	0.721	0.721
Positive regulation of blood coagulation	5	±	1	(4)	5	±	1	(4)	5	±	1	(3)	4	±	1	(3)	1.000	0.721	0.437
Negative regulation of hemostasis	7			(2)	7	±	1	(4)	6	±	2	(3)	6			(2)	0.182	0.495	-
Negative regulation of coagulation	7	±	1	(4)	7	±	1	(4)	7			(2)	6	±	1	(3)	1.000	0.633	0.352
Negative regulation of blood coagulation	6	±	1	(4)	6	±	1	(4)	6	±	1	(3)	5	±	1	(3)	1.000	0.721	0.721
Regulation of hemostasis	10			(2)	10	±	1	(4)	9	±	2	(3)	9			(2)	0.182	0.495	0.205
Regulation of coagulation	10	±	1	(4)	10	±	1	(4)	10			(2)	9	±	1	(3)	1.000	0.633	0.352
Regulation of blood coagulation	9	±	1	(4)	9	±	1	(4)	8	±	2	(4)	8	±	1	(4)	1.000	0.477	0.228
Hemostasis	15			(1)	15			(2)	15			(2)	12	±		(1)	-	0.667	-
Blood coagulation	13	±	2	(4)	15	±	1	(4)	12	±	3	(4)	13	±	2	(4)	0.228	0.585	0.766
Platelet activation				(-)	5	±	0	(3)				(-)	5			(1)	-	-	-

^1^ Gene ontology (analyzed by the STRING database); ^2^ number of positive samples.

**Table 6:** Urinary proteins associated with biological processes ^1^ related to other functional groups, detected in the urine of cats (*n* = 4) fed a high-protein diet without (w/o) or with the supplementation of arginine (Arg), ornithine (Orn) or zeolite (Ze). Mean number of proteins ± standard deviation.

Biological Process	w/o				Arg				Orn				Ze				*p*-value: w/o versus		
																	Arg	Orn	Ze
Positive regulation of extracellular exosome assembly	3	±	0	(4) ^2^	3	±	0	(4)	3	±	0	(3)	3	±	0	(3)	-	-	-
Iron ion transport	5			(1)				(-)	5			(2)	4			(1)	-	-	-
Regulation of body fluid levels	16	±	2	(4)	18	±	1	(4)	14	±	4	(4)	16	±	4	(4)	**0.039**	0.470	1.000
Negative regulation of hydrolase activity	24	±	1	(3)	26	±	2	(4)	25	±	1	(3)	25	±	1	(3)	0.366	0.492	0.349
Negative regulation of catalytic activity	29	±	2	(4)	30	±	3	(4)	29	±	1	(3)	26	±	7	(4)	0.401	0.538	0.513
Negative regulation of molecular function	32	±	1	(4)	33	±	4	(4)	32	±	1	(3)	32	±	2	(3)	0.462	0.900	0.832
Regulation of hydrolase activity	35	±	1	(4)	34	±	2	(4)	33	±	1	(3)	31	±	7	(4)	0.580	0.158	0.344
Negative regulation of multicellular organismal process	27	±	1	(4)	28	±	2	(4)	27	±	1	(3)	25	±	7	(4)	0.506	0.721	0.635
Regulation of catalytic activity	46	±	2	(4)	46	±	3	(4)	45	±	1	(3)	47	±	2	(3)	0.890	0.456	0.341
Regulation of multicellular organismal process	52			(2)	50	±	2	(3)	47	±	4	(3)	56			(2)	0.385	0.219	0.232
Regulation of biological quality	63	±	5	(4)	61			(2)	60			(2)	64	±	6	(3)	0.461	0.534	0.771
Negative regulation of biological process	78	±	5	(3)	76	±	5	(4)	71	±	4	(3)	79	±	3	(3)	0.523	0.119	0.780
Extracellular matrix organization	11	±	1	(3)	11	±	1	(4)	9	±	2	(3)	11	±	1	(3)	0.721	0.189	0.643
Biological adhesion	42			(1)				(-)				(-)				(-)	-	-	-
Positive regulation of transport	22			(1)				(-)	19			(1)	22			(2)	-	-	-
Interspecies interaction between organisms	31			(1)	26			(2)				(-)	27			(2)	0.212	-	-
Regulation of localization	50			(1)	44			(2)	44			(1)	48			(2)	0.084	-	0.454
Multicellular organismal process	107			(1)	94	±	4	(3)	36			(1)	76			(2)	0.106	-	0.592
ADP metabolic process	6			(1)				(-)				(-)				(-)	-	-	-
Regulation of protein-containing complex assembly	16			(1)				(-)				(-)	18			(1)	-	-	-
Generation of precursor metabolites and energy	12			(1)				(-)				(-)	13			(1)	-	-	-
Regulation of podosome assembly	3			(1)	3			(1)				(-)	3			(1)	-	-	-
Transition metal ion transport	7			(1)	7			(1)	7			(1)	7			(1)	-	-	-
Regulation of neurotransmitter receptor localization to postsynaptic specialization membrane				(-)	3			(1)				(-)	3			(2)	-	-	-
Response to toxic substance				(-)	9			(1)	8			(1)				(-)	-	-	-
Positive regulation of exosomal secretion				(-)	4			(1)				(-)	4			(1)	-	-	-
Zymogen activation				(-)				(-)	4			(1)	4			(1)	-	-	-
Negative regulation of metabolic process				(-)				(-)	42			(1)				(-)	-	-	-
Positive regulation of biological process				(-)				(-)	80			(1)	85			(1)	-	-	-
Cellular transition metal ion homeostasis				(-)				(-)				(-)	5			(1)	-	-	-
Reactive oxygen species metabolic process				(-)				(-)				(-)	7			(1)	-	-	-
Positive regulation of protein-containing complex assembly				(-)				(-)				(-)	11			(1)	-	-	-
Collagen-activated tyrosine kinase receptor signaling pathway				(-)				(-)				(-)	3			(1)	-	-	-
Locomotion				(-)				(-)				(-)	25			(1)	-	-	-

^1^ Gene ontology (analyzed by the STRING database); ^2^ number of positive samples.

**Table 7:** KEGG pathways ^1^ related to downregulated urinary proteins of cats fed a high-protein diet with an arginine supplementation compared to cats without a dietary supplementation.

Accession	Description ^2^	KEGG Pathway
XP_019667711.1	L-lactate dehydrogenase A chain isoform X2 [*Felis catus*]	Glycolysis/gluconeogenesis
XP_006933527.1	Triosephosphate isomerase [*Felis catus*]	Glycolysis/gluconeogenesis
XP_003996194.1	Beta-enolase [*Felis catus*]	Glycolysis/gluconeogenesis
XP_003994722.1	LOW QUALITY PROTEIN: aldehyde dehydrogenase, mitochondrial [*Felis catus*]	Glycolysis/gluconeogenesis
XP_003998682.1	Apoptosis-associated speck-like protein containing a CARD [*Felis catus*]	*Salmonella* infection
XP_003998706.1	Myosin regulatory light chain 2, skeletal muscle isoform [*Felis catus*]	*Salmonella* infection
XP_003982507.1	Actin-related protein 2/3 complex subunit 4	*Salmonella* infection
XP_011283864.1	Actin-related protein 2/3 complex subunit 2	*Salmonella* infection
XP_003992770.1	Small subunit ribosomal protein s3e; Ribosomal protein S3; Belongs to the universal ribosomal protein uS3 family (243 aa); 40S ribosomal protein S3 [*Felis catus*]	*Salmonella* infection

^1^ Kyoto Encyclopedia of Genes and Genomes (analyzed by the STRING database); ^2^ according to the STRING database and Proteome Discoverer Software 2.4.0.305.

**Table 8:** KEGG pathways ^1^ related to down- and upregulated urinary proteins of cats fed a high-protein diet with an ornithine supplementation compared to cats without a dietary supplementation.

Accession	Description ^2^	KEGG Pathway
**Downregulated urinary proteins**		
XP_003986621.1	Small subunit ribosomal protein s12e; Belongs to the eukaryotic ribosomal protein eS12 family (120 aa)	Ribosome
XP_003993856.1	60S acidic ribosomal protein P2 isoform X1; Belongs to the eukaryotic ribosomal protein P1/P2 family (117 aa)	Ribosome
XP_003996528.1	Large subunit ribosomal protein l23ae; Ribosomal protein L23a; Belongs to the universal ribosomal protein uL23 family (156 aa)	Ribosome
XP_003994766.1	Large subunit ribosomal protein lp0; 60S acidic ribosomal protein P0; Ribosomal protein P0 is the functional equivalent of E.coli protein L10 (317 aa)	Ribosome
XP_003992770.1	Small subunit ribosomal protein s3e; Ribosomal protein S3; Belongs to the universal ribosomal protein uS3 family (243 aa)	Ribosome; *Salmonella* infection
XP_011278541.1	RAB7A, member RAS oncogene family (207 aa)	*Salmonella* infection
XP_003998706.1	Myosin light chain, phosphorylatable, fast skeletal muscle (170 aa)	*Salmonella* infection
XP_011283864.1	Actin related protein 2/3 complex, subunit 2; Arp2/3 complex 34 kDa subunit; Functions as actin-binding component of the Arp2/3 complex which is involved in regulation of actin polymerization and together with an activating nucleation-promoting factor (NPF) mediates the formation of branched actin networks (377 aa)	*Salmonella* infection
XP_003982507.1	Actin-related protein 2/3 complex subunit 4; Functions as actin-binding component of the Arp2/3 complex which is involved in regulation of actin polymerization and together with an activating nucleation-promoting factor (NPF) mediates the formation of branched actin networks. Seems to contact the mother actin filament (186 aa)	*Salmonella* infection
**Upregulated urinary proteins**		
XP_003996904.2	Keratin, type i cuticular ha3-i; Uncharacterized protein; Belongs to the intermediate filament family (404 aa)	Estrogen signaling pathway
ABS11938.1	Trefoil factor family peptide 1; *Felis catus* trefoil factor 1 (TFF1), mRNA (81 aa)	Estrogen signaling pathway
XP_006940404.1	Keratin, type i cuticular ha1 isoform x3; Uncharacterized protein; Belongs to the intermediate filament family (409 aa)	Estrogen signaling pathway

^1^ Kyoto Encyclopedia of Genes and Genomes (analyzed by the STRING database); ^2^ according to the STRING database and Proteome Discoverer Software 2.4.0.305.

## Data Availability

The data set of the proteome analyses of this study is provided as a Supplementary Material.

## Supplementary Materials

The following supporting information can be downloaded at: https://www.mdpi.com/article/10.3390/vetsci9120654/s1, File S1: Urine proteome data set.
